# Rural Roma cultural heritage-based tourism in Central and Eastern Europe

**DOI:** 10.12688/openreseurope.21548.2

**Published:** 2026-01-14

**Authors:** Gábor Oláh, Eszter György, Boglárka Kőrösi, Patrik Mravik

**Affiliations:** 1 Atelier Department of Interdisciplinary History, Eötvös Loránd University, Budapest, 1088, Hungary

**Keywords:** Roma cultural heritage, cultural tourism, peripheral tourism, minority heritage, community-based tourism

## Abstract

This paper examines the intersection of Roma heritage, community development and tourism dynamics, with a particular focus on rural contexts, by exploring the areas of preservation, representation, promotion and potential economic development. This study forms part of a wider research initiative within the SECreTour project, which explores how cultural heritage can stimulate sustainable and equitable tourism development. A comparative, problem-oriented literature review focusing on Roma heritage-based cultural tourism initiatives in the CEE region is conducted to identify bottom-up initiatives, conceptual models, key heritage elements, and recommendations associated with Roma tourism offerings. In addition to examining these frameworks, the study maps top-down approaches by analysing European strategies, programs and projects targeting Roma heritage and tourism. Among the case studies examined, one pilot will analyse efforts to preserve and promote Roma heritage in Tomor and the surrounding villages located in a disadvantaged, peripheral region in North-eastern Hungary. These initiatives focus on various forms of intangible heritage, such as gastronomy, music and storytelling, as well as socio-cultural activities like participatory video production and extracurricular educational programs. Based on the collected data, the paper will establish an analytical framework for understanding Roma heritage tourism and its potential for sustainable community development.

## Background

### Introduction

This paper discusses an underexplored research topic that lies at the intersection of Roma heritage preservation, community initiatives, and rural development. While numerous studies in recent years have focused on the social and spatial integration of European Roma communities, less attention has been paid to heritage-making and preservation through cultural tourism, undoubtedly because few such initiatives are known to exist across Europe.

In recent decades, there has been a growing body of literature that examines the disadvantages faced by European Roma not only in the context of long-term social processes ( Powell & Lever, 2015: 2) and the resulting spatial and social segregation, but also explores issues related to the Roma's incomplete historical memory and cultural heritage. Going beyond the popular theory of the “art of forgetting” in the 1990, Van Baar (2011) states that there are more and more Roma communities who are “mobilizing memory as a strategy to inscribe Romani histories in the European memorial landscapes and to claim a place in national and European histories and memories.” ( Van Baar, 2011: 272).

In this article, we are looking at some specific examples of memory-making and heritagization in which Roma voices and cultural resilience are linked to bottom-up initiatives of cultural tourism.

### Roma heritage in the CEE region: invisibility, non-recognition and resilience

Central and Eastern European (CEE) countries’ aspiring tourism strategies faced a double challenge during the democratic and market economy transition after 1989–1990: 1) to (re)construct their national identities, and thus create tourism images liberated from communism, 2) to show eagerness to embrace European values. Culture and cultural heritage have become a primary means of creating a positive post-communist image, capable of representing national distinctiveness and blending into European openness. The unlocking of national identity discourses and the creation of a positive image through heritage have been built upon existing spatial, socio-economic and ethnic inequalities and accompanied by a harsh selective approach. Hence, certain social groups, ethnic minorities and geographical areas have been marginalized and excluded from the mainstream/majority narrative-based tourism image ( Hughes & Allen, 2005: 175).

As Jordan (2014) highlights, the drive to construct homogenized national narratives for tourism can lead to the neglect or reinterpretation of histories that do not align with this unified ethos, such as Roma heritage. Additionally, certain culturally significant sites or traditions may be valued for tourism without explicit acknowledgment of their association with specific social groups. However, Jordan also identifies counterexamples where minority heritage is actively appreciated, such as the recognition of Hanseatic, Prussian, and German heritage, as well as Jewish and Kashubian cultural traditions in Poland. It seems that minority heritage is more readily appreciated in CEE tourism when it pertains to a "national minority" rather than an "ethno-cultural minority." Although Roma communities in many CEE countries hold legal status as a recognized national minority (e.g., Hungary), this recognition remains incomplete or largely symbolic in several respects. This is evidenced by their limited cultural representation, the absence or underdevelopment of dedicated cultural institutions, and the overall marginalization of their historical and cultural visibility.

The "invisibility" of Roma culture and heritage is rooted in multiple layers of marginalization. Many Roma communities experience severe socio-economic disadvantages, including limited access to education, employment, and qualifications, often resulting in poverty or extreme poverty ( Breimo & Baciu, 2016; Kovács, 2015). Additionally, they predominantly reside in underdeveloped rural areas with inadequate infrastructure and low-quality housing conditions, as well as in segregated neighbourhoods. Beyond economic hardship, they also face ethnic exclusion and social stigma from the majority society, which can further deepen social conflicts and hinder their cultural recognition and representation.

Despite these obstacles to preserving heritage and preventing the loss of identity, various mechanisms and strategies have been observed that illustrate how Roma communities adapt their cultural practices to new environments while demonstrating resilience in the face of assimilatory pressures and policies ( Greenfields & Smith, 2018; György *et al.*, 2024). The openness and hybridity of cultural expressions play a crucial role in this process ( Silverman, 2014). The resilience of Roma groups stems from a combination of continuous adaptation and the retention of a degree of cultural autonomy. Okely (2012) argues that the Roma occupy a semi-autonomous, culturally hybrid space, in which they continuously create and recreate cultural autonomy while constructing authenticity through historical ingenuity and inventive originality in interaction with other cultures. Heritage, therefore, must be understood as part of both national and transnational narratives. Moreover, the role of local communities is essential in transmitting knowledge, leading to the emergence of hybrid spaces and initiatives where educational, social, and cultural practices intersect ( György & Oláh, 2018).

This paper is part of a broader research initiative within the SECreTour project ^1^, which seeks to demonstrate how cultural heritage can serve as a catalyst for sustainable and equitable tourism development while ensuring its preservation. Among the various European case studies examined, a pilot will analyse efforts to preserve and promote Roma heritage in Tomor and surrounding villages, located in a disadvantaged peripheral region of northeastern Hungary. These initiatives focus on diverse forms of intangible heritage, including gastronomy, music, and storytelling, as well as socio-cultural activities such as participatory video production and extracurricular educational programs. By synthesizing scholarly research, this paper explores cultural tourism initiatives, strategies, and opportunities centred on Roma heritage in the CEE region, particularly those that engage geographically peripheral areas or socio-economically and ethno-culturally marginalised communities.

### State-of-the-art in “placemaking” for Roma cultural heritage and tourism in CEE countries

This section examines the existing institutions dedicated to Roma culture and heritage, situating them within broader discussions on cultural heritage and memory-making processes with a comparative perspective in Central and Eastern European countries. The representations of Roma culture and history within heritage institutions remain limited. In particular, Roma-focused initiatives are notably scarce in the context of rural and peripheral cultural tourism activities. By reviewing the current landscape of Roma cultural institutions and their role in heritage and tourism, this section aims to provide a state-of-the-art on the debate on gaps, challenges and opportunities in the recognition and promotion of Roma heritage.

Liégeois (2013: 17) emphasises the intangible nature of Roma heritage, noting that it is defined more by movement and cultural expressions – such as language, social relations, music, and dance – than by physical monuments or built environments. While his analysis primarily concerns Western European traveler Roma communities, a similar perspective applies to the Roma heritage in the CEE region, where most of the communities have been sedentary for centuries. This intangible dimension has been acknowledged by international institutions such as UNESCO, which has included elements of Roma culture in its registers of intangible heritage and good practices ^2^.

Even though the Roma are the largest transnational minority in Europe, particularly present in the CEE countries, their cultural presence, institutions and civil initiatives related to the cultural and historical representation or heritage-making are primarily located in capitals and larger cities. Notably, the only major and representative Roma museum in all of Europe is located in Brno, Czechia. This institution, according to art historian Tímea Junghaus (2014: 31), is a “professionally installed museum space with multiple functions and a carefully elaborated strategy of presenting the history of Roma representation accurately and engagingly”. Since 2018, it has overseen the administration of the Roma Holocaust memorial at Hodonín u Kunštátu and Lety u Písku and since 2019, it has been also responsible for managing the Centre for the Roma and Sinti in Prague (CRSP). ^3^ Moreover, Roma culture as intangible cultural heritage comes up mostly in the context of urban festivals and guided tours in the 8th district of Budapest. As Zátori and Smith, 2015 state in their article, there are one or two initiatives outside Budapest with only few foreign tourists who are likely to venture them.

Elena Marushiakova and Vesselin Popov ( 2012; 2016) have published several overviews of international Roma museums and a recent doctoral thesis by Krisztina Varga (2024: 47) also examines examples of European Roma museums and cultural institutions. Varga provides a typology of these places according to their function, administrative structure and professional status. She identifies distinct categories, including standalone cultural history museums (e.g., the Museum of Romani Culture in Brno), branches of national museums (e.g., Slovak National Museum in Martin), ethnographic museums and their permanent exhibitions, as well as open-air ethnographic museums (e.g., Permanent Scandinavian Romani exhibition in Elverum, Norway), local history museums (e.g., Barcelona Urban Roma Eco-Museum), permanent exhibitions on Roma history (Museum of Personal Stories, Osijek, Croatia), and transnational institution (ERIAC, European Roma Institute for Arts and Culture).

Despite these examples, larger and more influential Roma cultural institutions remain absent from the European cultural landscape, particularly one dedicated to contemporary Roma art. While progressive Roma artists have gained visibility in major international exhibitions such as the Venice Biennale ^4^ or at the Documenta in Kassel ^5^, the economic resources and political will to establish a dedicated museum at the European level are still lacking. To address this gap, ERIAC has proposed RomaMoMA as a “contemporary art project initiating a forum for collaborative reflection on a future Roma Museum of Contemporary Art, with the involvement of local and international, Roma and non-Roma artists, cultural experts, social scientists and the civil sphere.” ^6^

Beyond the presence or absence of Roma museums, one may detect the increasing interest and focus on compensating the lack of traditional historiography and discovering and exhibiting Roma past and memory, often in relation to Roma holocaust remembrance ( Kelso & Eglitis, 2014; van Baar, 2011). The pioneer and exemplary project of the Tom Lantos Institute established a comprehensive database listing the location of Roma representations in the Hungarian capital and other parts of the country ( Bogdán *et al.*, 2023). The database comprehends monuments, memorial plaques, statues, wooden memorial columns, paintings and murals in public spaces, public buildings (named after Roma people) and public buildings (established by Roma people). This crucial research provides not only a unique collection of already existing successful and unsuccessful representations of Roma culture and identity but serves as a resource and a foundation for the creation of more representations of Roma culture and identity in public spaces. While probably the main theme of academic resources and activist projects still remain the remembrance and memory the Roma holocaust ( Kapralski, 2023; Stewart, 2004), ^7^ there are other examples in which public spaces are analyzed from the point of view of their Roma representation ( Bak, 2020; Gruber, 2009; Ruethers, 2013).

To summarize the arguments regarding the current status of Roma heritage and memory places, it can be observed that despite the relatively significant number of institutions and projects, these places continue to operate in a fragmented and hampered way. They often develop collections in parallel and remain highly dependent on the contingency of financial support. Moreover, beyond the few major museums and cultural centres, these sites are small-scale institutions, such as community centres or country houses, where the exhibition of Roma cultural heritage is integrated with broader cultural, educational, identity and community building, as well as social inclusive activities.

These spatially configured forms of intangible heritage, remembrance and cultural representation in public spaces are largely linked to top-down institutions, including EU-initiatives, national and regional governments and municipal authorities. However, one can observe that bottom-up initiatives aimed at preserving and valorising heritage, such as the one in Tomor in Hungary, occupy a distinctive position “somewhere” in the middle of this matrix. This “place-making” is an entirely bottom-up initiative, conceived and sustained by the local Roma community yet it maintains a broad network of partners and cooperations. Moreover, it stands as a rare example of Roma-led rural heritage preservation.

### Objectives

This paper aims to explore the intersection of Roma heritage, community development and tourism dynamics, particularly in rural contexts. The research is guided by the following key questions:

- Conceptual framework and models:
  ○ What concepts and models have emerged to describe the creation and management of Roma heritage tourism in rural settings?
- Heritage-based tourism initiatives:
  ○ How do top-down and bottom-up Roma heritage initiatives in Central and Eastern Europe embrace the development of touristic products and services?
  ○ What tangible and intangible heritage elements associated with Roma communities have emerged as potential resources for tourism enterprises?
- Marginalization and sustainability
  ○ How do spatial, socio-economic, and ethno-cultural marginalization shape the role of cultural heritage in fostering sustainable tourism and community development?

By addressing these questions, the study seeks to provide a deeper understanding of the dynamics between cultural heritage, tourism, and spatial, social and economic transformation within marginalized Roma communities.

## Method

To achieve the objectives outlined in this study and to provide an overview of the existing literature on an underexplored research topic, a comparative problem-oriented literature review is conducted, focusing on Roma heritage-based cultural tourism initiatives in the CEE region ^8^. This review is based on desk research and aims to identify bottom-up initiatives, conceptual models, key heritage elements and recommendations associated with Roma tourism offerings. Firstly, relevant scientific publications were identified between November 2024 and January 2025. Publications were retrieved from the following databases: Web of Science, ScienceDirect, Google Scholar and JSTOR. The search strategy employed the following keywords: Roma OR Gyps* AND tourism; Roma OR Gyps* AND heritage AND tourism; Roma OR Gyps* AND cultur* AND tourism. No publication date restrictions were applied. The geographical scope was limited to the CEE region and the Balkan countries, excluding former Soviet Union states. Only publications addressing non-metropolitan contexts – specifically rural, peripheral and small-town areas – were considered. Following this procedure, a total of 15 articles were identified. These publications cover the most recent decade, having been published between 2013 and 2024. The studies primarily focus on specific case studies from Hungary (4), Romania (4), Slovakia (2), Bulgaria (1), Croatia (1), Serbia (1) and Slovenia (1) as well as the Balkan region, with one study adopting a cross-border and macro-regional perspective.

In addition to examining grassroots frameworks, the study also maps top-down approaches by analysing European strategies, programs, and projects (Creative Europe, Framework Programmes) that target Roma heritage and tourism. This mapping activity provides a broader institutional and policy perspective on the development and promotion of Roma heritage-based tourism. Using relevant databases – namely CORDIS, the EU Funding & Tenders Portal and Creative Europe databases for ongoing and completed projects – seven projects were identified for the period of 2017-2026. These projects predominantly adopt transnational perspectives, typically involving partnerships between two and four countries. Only projects with a clear cultural or heritage orientation were included; consequently, broader objectives related to social inclusion or the reduction of inequalities were considered exclusively when they were addressed through cultural perspectives. Based on the collected data, the paper seeks to establish an analytical framework for understanding Roma heritage tourism, its dynamics, and its potential for sustainable community development.

However, this research faces certain limitations, primarily due to the underexplored nature of the field and language barriers. The study relies on sources available in English, which restricts access to relevant scientific articles, reports, and policy documents published in other national languages of the CEE region. This linguistic constraint may limit the comprehensiveness of the findings and the ability to incorporate locally produced knowledge.

Based on these identified resources, this study thus explores and analyses the intersection of Roma heritage and tourism, examining both preservation and representation alongside promotion and potential economic development. It seeks to identify the main conceptual models informing both theoretical approaches and practical applications of this intersection, as well as to examine which heritage elements have been valorised through cultural tourism initiatives and the recurring spatial settings, that is, the places where these initiatives have been implemented.

Neither of these aspects has been extensively established as socio-cultural practices or as subjects of academic inquiry. As a result, the preservation and (re)presentation of Roma heritage remain in a fragile state. This fragility significantly affects the analysis from a tourism perspective, given the scarcity of initiatives, persistent economic sustainability challenges, and the limited presence of targeted national or EU-level strategies and programs. Consequently, these factors also shape the availability of sources and data necessary to map the landscape of Roma heritage tourism in the CEE region.

## Results

### Roma heritage and tourism in European strategies and projects

Recently, EU-level research and innovation initiatives, along with educational, cultural and creative programs – particularly the EU framework programs, Erasmus+ and Creative Europe – have increasingly focused on the multicultural character and linguistic diversity of the CEE region, including its minority heritage ( Banaszkiewicz *et al.*, 2017: 5). However, the EU Roma Strategic Framework before 2020 was widely criticized for its omission of arts, culture, and history, which received only a brief mention under the educational curriculum. This exclusion represented a critical gap, as the underrepresentation of Roma heritage at the national level has been perpetuated within the broader European narrative. This approach framed the disadvantaged situation of the Roma population as a series of closely interrelated social and economic problems, excluding cultural rights from both problem identification and potential development opportunities. One symbolic manifestation of this issue is the scattered and unorganized collections of Roma artworks, often stored away in storerooms and basements ( Matarazzo & Naydenova, 2019: 18–19). Nevertheless, the new Framework (2020–2030) has recognized culture and history as levers for addressing socioeconomic inequalities ( EC, 2020). Despite this, access to resources for preserving Roma heritage remains severely limited, and tourism is notably absent from the identified development opportunities.

If explicitly targeting Roma communities, EU research and innovation (R&I) projects tend to focus more on issues of social cohesion, political rights, and historical traumas, with comparatively less emphasis on heritage. This trend was highlighted in a mapping exercise conducted as part of the REACH project ( György *et al.*, 2020: 8). The review of EU-funded projects revealed that, while several initiatives addressed aspects of cultural diversity and the heritage of marginalized communities, none specifically focused on the cultural heritage of Roma communities. Since the review of the R&I projects, research on Roma heritage has focused on participatory approaches (REACH), cultural valuation practices at the local level (UNCHARTED) and the digitization and documentation of Roma art and heritage under the framework of Europeana (WEAVE).

Comparatively, more projects have emerged focusing on creativity, culture, and educational programs. Most of the projects launched in the framework of these programs were designed to promote the recognition and knowledge of Roma cultural heritage. Recurrent themes of the projects include the empowerment and education of marginalized adolescents, community building, the overcoming of isolation and the promotion of social inclusion through culture, arts and research. As discussed above, these elements have the potential to contribute to the development of tourism.

ROMHERITAGE ^9^, which ran from 2023 to 2024, sought to establish Romani heritage itineraries and link neighbourhoods, sites, museums and research in Spain, Italy and Slovenia in order to make visible the most significant tangible and intangible manifestations of Romani culture. “Roma Soul Food” (ROMASOUL) ^10^, an ongoing project, also aims to build transnational links between artists, activists and researchers through heritage, music and oral culture. Established between Serbia, Greece and Slovenia, the project intends to combine music and cooking by building a digital audio-visual archive of culinary recipes in Romani language and its variants, as well as recording a music album. In the past 5 years, a series of projects have been initiated, each with a focus on storytelling, theatre and performativity. Completed in 2022, the “European Roma Theater - Contemporary Cultural Heritage Shapes Our Future” project ^11^ was coordinated by a Hungarian civil organization, in collaboration with partners from Austria, Ukraine and Romania. The project’s primary objective was to nurture a sense of unity among distinct European Roma theatres, involving a network of 16 European countries. It facilitated the creation, translation and performance, as well as the video recording of narratives that shed light on both the challenges and values of various Roma groups. Currently funded by Erasmus+, the Hungarian coordinator has expanded the project under the title “European Roma Young Theater Artists and Facilitators 4 Inclusion and Empowerment (ERYTAF4IE)” ^12^, in partnership with organisations from Czechia, Italy, Romania and Slovakia. The mission of the international non-formal educational program is to continue enabling mutual learning through drama and performance. Parallel to this program, with the coordination of the Romanian partner and the participation of the Hungarian and Italian partners, the Creative Europe-funded „Stand Up Roma” (SUR) ^13^ has been launched. The focus of the project is to employ humour as an instrument within personal storytelling, with the intention to stimulate critical thinking and social inclusion. Similarly to the projects discussed above, the recorded plays and performances will be archived in the Roma Heroes Digital Collection of European Roma Theater and Drama ^14^.

As with ROMHERITAGE and ROMASOUL, a core aspect of the theatrical projects has been, and continues to be, transnational mobility and visibility, in addition to the strengthening of identity and resilience. The discovery and presentation of talent and values in domains such as the performing arts, music, culinary arts, and various forms of tangible and intangible heritage, combined with the processes of transnational network-building, the involvement of new audiences, as well as digitalization and self-archiving, challenges the notion that Roma culture is either isolated or segregated: Indeed, the overarching goal of the projects is to integrate Roma culture into mainstream realms of creative and cultural production, ultimately locating it in the broader context of multicultural Europe. This aspect is crucial in positioning Roma heritage – rural and urban alike – within the broader cultural landscape, thus facilitating the utilization of its touristic potential.

Roma heritage primarily appears in cultural, creative, and educational projects, with less representation in research and innovation. The few identified projects highlight a broader trend in which public subsidies and international targeted grants play a crucial role in initiating and sustaining activities. However, there is less emphasis on heritage and cultural tourism as either an objective or a strategic lever ( György *et al.*, 2020).

### Tourism models for representing Roma heritage

When defining the type of tourism associated with Roma cultural representation as well as heritage-based initiatives, several concepts and models emerge in the relevant literature. However, they seem to be largely strategic and programmatic in nature, rather than being descriptive or interpretive of an existing initiative. This section examines these models that conceptualize and attempts to operationalize tourism initiatives within the realm of Roma heritage, providing a framework for understanding and analysing their characteristics and broader impact. It also explores how these initiatives fit within the broader literature on sustainable cultural tourism.

In the literature on sustainable cultural tourism, *community-based tourism (CBT)* frequently emerges as both a theoretical framework and a practical model. Rooted in the (re)activation of community bonds, empowerment, and participation ( Corsale & Iorio, 2014), CBT is not only ecologically sustainable but also economically viable, as well as ethically and socially equitable ( Havadi-Nagy & Espinosa Segui, 2020: 144–145). Havadi-Nagy and Espinosa Segui (2020) emphasize that CBT is highly versatile, allowing application across a wide range of contexts, particularly in minority communities. Inclusivity is ensured through the recognition of heterogeneity within the community, which can be addressed by identifying shared values, common good or cultural heritage. However, CBT is often criticized for being idealistic or romanticized. In this context, the role of local leaders ( Corsale & Iorio, 2014) or “individual heroes” ( György & Oláh, 2018) become crucial in fostering empowerment, mobilizing resources and building stakeholder network, highlighting the significance of individual leadership in the implementation of CBT.

Much of the literature on sustainable cultural tourism has evolved around the spatial redistribution of visitor flows, shifting from metropolitan centres to the peri-urban or rural areas, from mass tourism to small-scale destinations, from standardized offerings to personalized and differentiated products and services. Within this framework, rural Roma heritage tourism activities can be understood as a form of *village tourism* or *ecotourism* ( Kantar, 2023; Szente *et al.*, 2021) or more precisely, as a type of *niche tourism* or *special interest tourism* ( Sorea & Csesznek, 2020). Unlike large-scale tourism, these approaches emphasize personalized and unique experiences. Such special interest tourism practices are particularly relevant in the contexts of *heritage tourism* and *ethnographic tourism,* both of which have been extensively studied in rural areas of the CEE region ( Hutárová *et al.*, 2021). In this type of tourism, living and built elements of culture and folkways, thus tangible and intangible heritage elements may be part of of the offer, such as monuments, historic public buildings and homes, farms but music, dance, language, religion, folkways and cuisine, artistic traditions, and festivals as well ( Sorea & Csesznek, 2020: 3–4). The valuation of cultural heritage in this domain has particular importance as it often means the recognition of heritage practices endangered by rural exodus and urbanization processes. As some Romanian examples showed, the early realization of the valuable cultural heritage may be crucial before major interventions in the buildings and environment would spoil the village scape and ruin a significant structure and foundation for unfolding rural tourism ( Havadi-Nagy & Espinosa Segui, 2020: 160).

In the context of Roma communities and heritage, the concept of *ethnic tourism* provides a valuable framework for understanding tourism dynamics. This type of tourism is motivated by the aspiration to experience and engage with the distinctive cultural practices, lifestyles and heritage of ethnic, minority or indigenous groups. It is characterized by an emphasis on traditionalism, cultural authenticity and the “exoticism” of these groups ( Hutarova & Kozelova, 2021). Pal and Nadda (2018) argue that the development of Roma ethnic tourism has a significant role to develop tourism, bringing both positive and negative impacts. While it can generate potential positive socio-economic benefits, including job opportunities, improved infrastructure, and enhanced living standards for local communities, it also presents challenges. Increased tourism may disrupt balance of living, introduce foreign cultural influences that alter authenticity of Roma heritage, and create difficulties in maintaining heritage practices due to tourist presence. Due to these controversies, the academic discourse on ethnic tourism addresses ethical issues regarding ethnic tourism, such as social stigma, stereotypes, poverty and marginalization ( Hutarova & Kozelova, 2021).

An alternative model that could be evaluated and adapted to the analysis of Roma heritage tourism is the concept of *pro-poor tourism*. Emerging primarily in the 1990s, this term was originally used in reference to developing countries of Africa and Asia. However, it has been widely criticized for being pejorative and problematic. As a result, alternative terms, such as *anti-poverty tourism* or *tourism as a tool for poverty alleviation, reduction* or *elimination* have been introduced in the literature ( Matlovicová *et al.*, 2022: 2–3). The support of poor communities through the tools of sustainable development of tourism may be highly relevant in the research of the Roma communities, especially because of previous research on marginalized Roma communities in Slovakia and their potential involvement in cultural touristic activities ( Hutarova & Kozelova, 2021; Matlovicová *et al.*, 2022).

Within the framework of sustainable cultural tourism, several EU policies focus on promoting *transnational tourism* as a means of diversifying tourism offerings. Among the various products and services, itineraries and cultural trails have been proposed to connect independently established sites, highlighting the interconnected and fluid nature of cultural boundaries that transcend national borders. This type of tourism aligns well with the development of a network of Roma heritage places and initiatives, leveraging the transnational character of Roma communities. International initiatives (e.g., ROMHERITAGE) have already been launched to test and implement cultural routes through pilot projects, aiming to establish a cohesive network of otherwise fragmented and sporadically located Roma heritage initiatives. These efforts are in line with broader international projects dedicated to collecting, documenting, promoting, and disseminating elements of Roma cultural heritage (e.g., WEAVE). In this context, the role of ERIAC should be particularly emphasized.

### A landscape of bottom-up placemaking for Roma heritage and tourism

After reviewing the different types of tourism associated with Roma heritage, this section examines the manifestations of Roma heritage tourism by exploring intangible heritage practices that have been transformed into tourism offerings. It identifies spaces where tangible heritage elements and intangible heritage practices are represented and presented, thereby spatializing Roma heritage. It explores how Roma communities create physical and digital spaces for heritage, as well as the ways in which activities are organized around these sites. In doing so, it investigates the processes of bottom-up placemaking. Specifically, it addresses the question: In what types of spaces does Roma heritage take shape in the CEE region?

The ROMHERITAGE project itinerary in Slovenia, which includes both urban and rural sites, highlights key locations where Roma heritage is presented in CEE countries through temporary programs and permanent sites. Temporary programs primarily consist of performing arts shows, festivals and exhibitions, often hosted in local cultural and educational centres. In contrast, permanent sites focus on ethnographic heritage and the historical everyday living conditions of Roma communities, which may be represented in entire villages, open-air museums (skansens), restaurants, country houses or community centres. This section explores other manifestations of such sites in the CEE regions and highlights their operational characteristics.

Viscri, a multi-ethnic Romanian *village* and a UNESCO World Heritage site, is predominantly inhabited by Roma and has become a well-known and distinctive example of CBT. The village sustains a high volume of tourism, primarily showcasing Saxon architectural heritage. A key external figure in the restoration of its architectural heritage has been the Prince of Wales, now King Charles III of the United Kingdom. Community members have been empowered to diversify tourism offerings, including artisan crafts, local food products, hospitality, and catering services. However, Viscri presents an ambivalent case for Roma heritage. The discourse surrounding its heritagization primarily frames it as a “Saxon village”, selectively highlighting Transylvanian Saxon cultural elements while shaping the tourism narrative around Saxon heritage. Consequently, Roma and even Romanian everyday practices and heritage remain largely overshadowed ( Corsale & Iorio, 2014).

The Roma *settlement* Kamenci, located in Crenšovci in the Prekmurje region of northwestern Slovenia, lies near the Hungarian, Austrian and Croatian border. It has transformed Roma heritage into a cultural tourism attraction, integrating tourism into a broader development strategy aimed at fostering social inclusion. Kamenici can be categorized as a heritage village or an open-air ethnographic museum. It has been recognized as a good practice within the “Cultural Resources for Roma Inclusion” project by the Council of Europe and the EU as an initiative that could inspire other initiatives or be adopted to other contexts. In 2003, Kamenci opened Slovenia’s first Roma museum, which presents the historical everyday life of the community, oral traditions, local legends, traditional crafts, and botanical products historically used in local medicinal practices. In the 2010s, a community center was established to support Roma social entrepreneurship in the field of cultural tourism. These initiatives have been developed in collaboration with Roma and non-Roma associations, the local municipality, and active participation from the community ( Sardelic & Sardelic, 2013).

*Roma country houses*, which showcase rural Roma traditions, ethnographic heritage, and everyday living conditions - encompassing both tangible and intangible heritage - can be found in various locations. Notable examples include those in Hodász, North-Eastern Hungary ^15^ and in Maglenca, Central-Croatia ^16^. The latter was researched in the frame of an INTERREG project ^17^, focusing on life-long learning programs for developing ecotourism. Analysing the results of this project, Szente *et al.* (2021), state that the possibility of including the Roma population in ecotourism is generally very limited because of underlying operationalization problems of their inclusion, such as the government’s weak support. They cite the Touristic Destination Management in Somogy County, Hungary, who, when asked about Roma-based touristic and community initiatives, seemed not to have “any information at all about what this social group could offer or could be capable of contributing to tourism” (236). This lack of interest or rather hostile attitude is complemented with the high level of segregation of marginalized Roma communities, which stands as a fundamental barrier to the implementation of cultural-, or community-based tourism practices ( Matlovicová *et al.*, 2022: 4).

A recent research looked at the potentials of pro-poor tourism principles toward Roma communities living in the central part of the Spiš region in Slovakia to see if, in the immediate context of tourist destinations, Roma communities may be better involved in the touristic offers than before. They assess several municipalities in relation to their cultural-historical potentials, their human resources (looking at the rate of Roma unemployment, the proportion of Roma involved in ‘activation works’ (state-subsidized work for the poor, which was organized by municipalities, similar to the Hungarian public work scheme ^18^) and the specific potential for the development of Roma culture, that is to say, the existence of *community centres*, the operation of NGOs, citizens’ associations or any Roma cultural, artistic, and economic activities, active Roma craftspeople/artists ( Matlovicová *et al.*, 2022: 5). The results of the study show that despite individual and local differences, there are some common findings: on the one hand, the existence of community centres, offering leisure activities, especially for Roma children and youth, educational training and courses; dancing, singing or art clubs, handicraft and cooking clubs, etc., are crucial, because of their diversity and their interpretation as open spaces for training and developing the skills needed for tourism development. These centres, according to the authors, may initiate projects to obtain grant funding to support activities related to the tourism sector. On the other hand, the safeguarding and development of Roma culture and heritage practices should form the core of the tourism product ( Matlovicová *et al.*, 2022: 12–13). Both criteria appear in the case of Tomor: a home restaurant was established by locals in 2015 and, while it has multiple functions, most of the time the focus is not on gastronomy, but on being a community space for local youth. Plus, the main “products” that are promoted for tourists are traditional Roma meals that pass on the knowledge and recipes of previous generations.

Gastronomic heritage plays an important role in the development of rural tourism. It may be seen as an additional value in the tourism sector that serves as a means for attracting tourists and represents a territorial capital, providing social, ecological, and economic benefits as well. As authenticity is interpreted as a universal value and the driving force of the tourist movement, gastronomy may be relevant in the quest for authentic and traditional cultural experiences. In the case of ethnic minorities, gastronomic exposure may also be significant as a tool to learn about different national cuisines, pointing out the authenticity of the ancestors’ recipes ( Kalenjuk Pivarski *et al.*, 2023: 3403–3413). There are examples of restaurants specialising in Roma cuisine, mainly located in urban areas, such as Romani Kafevna in Maribor, Slovenia. As a form of intangible heritage, Roma cuisine is represented in different local culinary events, including folklore festivals and culinary exhibitions in Bulgaria ( Ivanova & Krastev, 2023: 147).

In addition to location-based cultural tourism, initiatives have emerged to promote mobile tourism experiences. Examples include Tomor’s Romama restaurant and a mobile buffet (food truck) featured at festivals and urban venues, which combine gastronomy with live music.

The digital space has become a crucial platform for documenting and presenting Roma heritage, recognized by both EU strategies and institutions, as well as grassroots initiatives, for its significant potential. At the European level, notable examples include ERIAC’s RomArchive, the Europeana sub-collection curated through the WEAVE project, and the Roma Heroes Digital Collection of European Roma Theater and Drama. Additionally, numerous local initiatives have embraced digital tools to document Roma heritage, often generating vast collections of audio recordings, videos, and photographs from local communities. However, these materials are frequently unstructured, posing a risk of loss if not properly archived and preserved.

## Conclusion

The issues presented in this paper align with the broader challenge of fostering sustainable tourism and community development in marginalized contexts. They shed light on the dynamics between community building, local identity construction, tourism initiatives, and local economic development. A key question that emerges is how community needs and expectations are integrated into tourism development. The examples presented illustrate how Roma heritage tourism serves as a unifying space for diverse community-based activities, incorporating a mix of intangible heritage elements to address multiple forms of marginalization.

The sporadically located bottom-up initiatives aimed at preserving and managing Roma heritage as “living heritage” often integrate a wide range of activities, from community-based programs to educational programs for multiple generations. The inherently complex and multifaceted nature of these initiatives makes it difficult to separate their social, cultural, and economic objectives. This is largely due to the systemic disadvantages faced by Roma communities across various aspects of societal life, as well as the explicit mission of fostering social cohesion through culture and education. In this context, the possibility of tourism also emerges, not only for its potential to drive local economic development but, more importantly, for its role in fostering community connectedness and transgenerational knowledge transfer. Thus, tourism is not articulated as an objective but a lever for broadening young people's life perspectives and facilitating the effective transmission of knowledge across generations. All of these elements converge in the concept of heritage, which serves as a catalyst for managing change, a means of strengthening social bonds, and ultimately a safeguard for the sustainability of both the community and its initiatives.

When applying these insights to the Romama in Tomor, a pilot site within the SECreTour project, there are valuable lessons to be drawn from existing practices in Roma heritage tourism. A key observation is that the community itself serves as both the foundation and the objective of this initiative. The sense of belonging is reinforced and sustained through video production and the support of the Romama Social Cooperative umbrella organisation. Roma identity is intentionally represented in a positive light, with heritage serving as an effective means of fostering this representation. However, the Cserehát subregion, including Tomor, is not traditionally positioned as a tourist destination and lacks support from government or municipal actors. Despite these challenges, developing the Romama home restaurant and other tourism-related initiatives is not an impossible endeavour, it presents an opportunity for strategic development. One of the first crucial steps is identifying and getting inspiration from successful models from existing good practices. In this process, research plays a crucial role, not only in documenting and analysing these initiatives or even co-creating participatory heritage research but also in mediating between grassroots community efforts and policy development.

These studies face considerable limitations. Among other similar cases, Tomor is located in an area facing social and geographical challenges. In terms of public transport and infrastructure, Tomor does not benefit from the meagre resources of the already depressed North-Eastern region. The temporary nature of funds and collaborative research projects results in the limitation of time and the possibility of strategic planning. The asymmetric relationship between the professional research institution and the local community, poses a further difficulty. The limited literature on Roma cultural heritage and tourism also highlights a significant research gap, particularly regarding marginalized and peripheral Roma communities in Central and Eastern Europe. This paper has aimed to address this gap by synthesizing existing studies on the relationship between Roma communities, their heritage and cultural tourism. The scarcity of research can be attributed to everyday discrimination and stigmatization that Roma communities face across Europe from various social and cultural perspectives. Moreover, when considering the potentials of cultural tourism in rural areas of the CEE region, Roma communities experience even more radical marginalization due to higher unemployment rates and lack of opportunities. Nevertheless, some examples indicate that the integration of Roma communities and their cultural heritage into cultural and ecotourism offerings has already begun to emerge sporadically across most countries in the region, highlighting the need for further research and policy support to enhance the recognition of Roma heritage and foster a more inclusive and sustainable approach.

The comparative overview of Roma heritage tourism practices can contribute to the discourse on culture as a community-building tool and economic self-sustainability. Facing similar challenges in the CEE region, different forms of Roma heritage initiatives could serve as instructive practices to learn from. To illuminate the cultural values of Roma communities, participatory heritage research might be a potential path for future research. Speaking with, rather than about, the community could be a tool for breaking down existing stereotypes. A collaborative way aims to help to discover their own cultural heritage. The case study of Tomor shows that cultural heritage can serve as a tool for empowerment, and the commonly explored cultural values and skills as self-representation.

## Ethics and consent

## Data Availability

No new data were created or analysed during this study. Data sharing is not applicable to this article.

## Footnotes

^1^ Funded by the European Union. Views and opinions expressed are however those of the authors only and do not necessarily reflect those of the European Union or European Commission. Neither the European Union nor the granting authority can be held responsible for them. SECreTour *(Sustainable, Engaging and CREative Tourism as a driver for a better future in rural and remote areas)* is a three-year research and innovation project under the Horizon Europe framework programme, running from 2024 to 2027.

^2^ As an example, the UNESCO-backed national inventory of Intangible Cultural Heritage in Hungary includes one element that is related to Roma heritage, the Hungarian and Gypsy Dance Traditions of Nagyecsed. Additionally, there are two elements under the National Elements of Good Safeguarding Practices: the Rajkó method and the Pedagogic Method of the Gandhi Institute in Pécs. (Intangible Cultural Heritage in Hungary. Retrieved 3 March 2025, from https://szellemikulturalisorokseg.hu/index_en.php).

^3^ Museum of Romani Culture. Retrieved 19 February 2025, from https://www.rommuz.cz/en/museum/about-us/

^4^ Paradise Lost: The First Roma Pavilion at the Venice Biennale. (2007). Retrieved 4 March 2025, from Open Society Foundations website: https://www.opensocietyfoundations.org/publications/paradise-lost-first-roma-pavilion-venice-biennale

^5^ One Day We Shall Celebrate Again: RomaMoMA at documenta fifteen. (2022, June 6). Retrieved 4 March 2025, from ERIAC website: https://eriac.org/one-day-we-shall-celebrate-again-romamoma-at-documenta-fifteen/

^6^ https://eriac.org/what-is-romamoma/

^7^ https://www.roma-sinti-holocaust-memorial-day.eu/education/verortungen/ https://dokuzentrum.sintiundroma.de/en/education/exhibitions/permanent-exhibition-heidelberg/

^8^ In this study, Central and Eastern Europe (CEE) refers specifically to Central European and Balkan post-socialist countries, excluding those that were part of the former Soviet Union.

^9^ European Itineraries of Roma Cultural Heritage (ROMHERITAGE), Creative Europe, ref.: 101100263, 2023–2024 (24 months) https://culture.ec.europa.eu/creative-europe/projects/search/details/101100263

^10^ Roma Soul Food (ROMASOUL), Creative Europe, ref.: 101174182, 2024–2026 (24 months) https://ec.europa.eu/info/funding-tenders/opportunities/portal/screen/opportunities/projects-details/43251814/101174182/CREA2027?keywords=Roma&isExactMatch=true&order=DESC&pageNumber=1&pageSize=50&sortBy=title

^11^ European Roma Theater - Contemporary Cultural Heritage Shapes Our Future, Creative Europe, ref.: 607436-CREA-1-2019-1-HU-CULT-COOP1, 2019–2022 (20 months). https://culture.ec.europa.eu/creative-europe/projects/search/details/607436-CREA-1-2019-1-HU-CULT-COOP1

^12^ European Roma Young Theater Artists and Facilitators 4 Inclusion and Empowerment (ERYTAF4IE), Erasmus+, ref.: 101185876, 2024–2026 (24 months). https://ec.europa.eu/info/funding-tenders/opportunities/portal/screen/opportunities/projects-details/43353764/101185876/ERASMUS2027?keywords=Roma&isExactMatch=true&order=DESC&pageNumber=3&pageSize=50&sortBy=title

^13^ Stand Up Roma (SUR), Creative Europe, ref.: 101173693, 2024–2026 (24 months). https://ec.europa.eu/info/funding-tenders/opportunities/portal/screen/opportunities/projects-details/43251814/101173693/CREA2027?keywords=Roma&isExactMatch=true&order=DESC&pageNumber=1&pageSize=50&sortBy=title

^14^ Roma Heroes - Digital Collection of European Roma Theater and Drama. Retrieved 19 February 2025, from https://romaheroes.org/

^15^ *Roma country house in Hodász.* Retrieved 19 February 2025, from https://www.romatajhaz.hu/

^16^ *Romany house Maglenča*. (n.d.). Visit Bjelovar-Bilogora County. Retrieved 19 February 2025, from https://bjelovar-bilogora.croatia.hr/en-gb/romany-house-maglenca

^17^ *Accepted financing of projects within 1st call Interreg Hungary Croatia*. (2017, July 3). Koprivnica Križevci Country. https://arhiva.kckzz.hr/en/prihvaceno-financiranje-projekata-u-sklopu-1-poziva-interreg-madarska-hrvatska/

^18^ Rorke, B. (2019, June 14). *Road to nowhere for Hungary’s Roma: Public works scheme is futile and insidious*. European Roma Rights Centre. http://www.errc.org/news/road-to-nowhere-for-hungarys-roma-public-works-scheme-is-futile-and-insidious
