# Supplementary material for: Rural Roma cultural heritage-based tourism in Central and Eastern Europe
Source: Open Res Eur. 2026 Jan 14;5:340. Originally published 2025 Nov 6. [Version 2] doi: 10.12688/openreseurope.21548.2 (PMC12811716; doi:10.12688/openreseurope.21548.2)
Supplement: Peer review history

# Reviewer report: Reviewer response for version 2

**DOI:** 10.21956/openreseurope.24548.r68068

**Published:** 2026-01-16

**Authors:** Remus Creţan

**Affiliations:** 1 West University of Timișoara, Timișoara, Romania

The paper is much improved, so it can be accepted for indexing.

If any, are all the source data and materials underlying the results available?

Yes

Is the work original in terms of material and argument?

Yes

Is the work clearly and cogently presented?

Yes

Is the argument persuasive and supported by evidence?

Yes

Does the research article contribute to the cultural, historical, social understanding of the field?

Partly

Does it sufficiently engage with relevant methodologies and secondary literature on the topic?

Yes

Reviewer Expertise:

Human Geography

I confirm that I have read this submission and believe that I have an appropriate level of expertise to confirm that it is of an acceptable scientific standard.

# Reviewer report: Reviewer response for version 1

**DOI:** 10.21956/openreseurope.23310.r63772

**Published:** 2025-11-21

**Authors:** GURGU ELENA

**Affiliations:** 1 Spiru Haret University, Bucharest, Romania

**Originality:**

 The paper presents a relevant and timely topic with a good level of originality. The focus on Roma cultural heritage and tourism is valuable and contributes to an under-researched area.

**Relevance:**

 The manuscript is highly relevant to cultural heritage studies, minority inclusion, tourism development, and Central/Eastern European socio-cultural research.

**Methodology:**

 The methodology section requires an improvement. The research design, data sources, and analytical approach are not clearly defined or sufficiently explained.

**Clarity and Structure:**

 The paper is generally understandable but would benefit from a clearer structure, more coherent argumentation, and better alignment between objectives, analysis, and conclusions.

**References:**

 The reference list is extensive, diverse, and up-to-date, demonstrating strong engagement with existing scholarship. However, the paper would benefit from integrating these sources more analytically rather than descriptively.

**Depth of Analysis:**

 Analysis remains largely descriptive. The manuscript should develop more critical insights and stronger conceptual or theoretical contributions.

**Contribution to the Field:**

 The topic has clear potential for meaningful scholarly contribution, especially regarding Roma representation and heritage, but the current version requires a deeper analytical development to fulfil that potential.

**Language Quality:**

 The language is generally good, with only minor issues in flow and cohesion .

**Overall Recommendation:**

 Revisions are needed, particularly in strengthening the methodology, improving structure, and deepening the analytical dimension.

If any, are all the source data and materials underlying the results available?

Partly

Is the work original in terms of material and argument?

Yes

Is the work clearly and cogently presented?

Partly

Is the argument persuasive and supported by evidence?

Partly

Does the research article contribute to the cultural, historical, social understanding of the field?

Yes

Does it sufficiently engage with relevant methodologies and secondary literature on the topic?

Partly

Reviewer Expertise:

management, heritage, culture, ethics, law

I confirm that I have read this submission and believe that I have an appropriate level of expertise to confirm that it is of an acceptable scientific standard.

# Response

**Published:** 2026-01-06

**Authors:** Gábor Oláh

- The ***Methodology*** section has been revised. The research design, data sources, and analytical approach are now more clearly defined. The section specifies the search strategy, keywords, databases used, types of documents analysed, and the approach to data interpretation. In addition, we reflect on the advantages and limitations of the data, as well as the temporal and geographical scope of the analysed literature.
- The **overall structure** of the manuscript has been improved to ensure better coherence and alignment between research objectives, analysis, and conclusions. The revised *Introduction* clarifies the study’s aims, the *Methodology* explains how these aims are addressed, and the *Conclusions* explicitly link findings back to the stated objectives.
- While the reference list was already extensive, we have revised the conceptual background. The *Introduction* now engages more critically with key debates in Roma studies, cultural heritage, and heritage tourism, including scholarship from Central and Eastern Europe.
- The analysis has been strengthened with more critical reflection and conceptual discussion. The *Conclusions* section now explicitly outlines the study’s theoretical contributions, practical implications, and how it advances understanding of Roma representation and heritage-making.
- The manuscript now more clearly positions its contribution within broader debates on minority inclusion, heritage and tourism. The *Conclusions* also identify avenues for future research that can build on the paper’s findings.
- Minor **language and stylistic revisions** have been made throughout the manuscript to improve clarity.

# Reviewer report: Reviewer response for version 1

**DOI:** 10.21956/openreseurope.23310.r64419

**Published:** 2025-11-20

**Authors:** Remus Creţan

**Affiliations:** 1 West University of Timișoara, Timișoara, Romania

This is an interesting study on rural Roma cultural heritage-based tourism in Central and Eastern Europe. The study has nice empirical findings, but there are some issues which could be revised and/or expanded.

 First, the introduction should more clearly state what this study brings new in international Roma studies and how exactly this study pushes forward what we know.

 Second, the conceptual background could be expanded and this is reflected also in the short reference list of the paper. So authors have to include more studies on broader Roma issues and case studies in different countries of Central and Eastern Europe (see for instance Powell Ryan's studies on Roma, and even other studies on Roma in Hungary) as well as more studies on heritage tourism and their link to the Roma areas.

 Third, the methodology is nicely presented, but advantages and limitation of data and method should be also addressed. Also, authors mentioned that some documents are analysed, but readers need to know what is the name of those documents and how data interpretation was made.

 Fourth, results are the strenghts of this study. 

 Finally, conclusions should be expanded by addressing also the theoretical and practical implications of this study, how other future studies could develop the ooutcome of this paper as well as the broader limitation of this research.

If any, are all the source data and materials underlying the results available?

Yes

Is the work original in terms of material and argument?

Yes

Is the work clearly and cogently presented?

Yes

Is the argument persuasive and supported by evidence?

Yes

Does the research article contribute to the cultural, historical, social understanding of the field?

Partly

Does it sufficiently engage with relevant methodologies and secondary literature on the topic?

Yes

Reviewer Expertise:

Human Geography

I confirm that I have read this submission and believe that I have an appropriate level of expertise to confirm that it is of an acceptable scientific standard, however I have significant reservations, as outlined above.

# Response

**Published:** 2026-01-06

**Authors:** Gábor Oláh

- The ***Introduction*** has been revised to clearly state the article’s original contribution to international Roma studies. It now specifies the study’s objectives and highlights its focus on Roma’s incomplete historical memory and cultural heritage, with particular attention to bottom-up cultural heritage initiatives as strategies for reclaiming Romani histories within European memorial landscapes. This emphasis addresses an underexplored area in the literature and clarifies how the article advances existing scholarship.
- The **conceptual background **has been expanded in the *Introduction*. We have incorporated additional scholarship on Roma studies, including the recommended author.
- The ***Methodology*** section has been revised to address the advantages and limitations of the data and methods used. It now provides a more detailed description of the document analysis, including the search strategy, keywords and databases used to identify relevant literature. In addition, the section reflects on the temporal scope and geographical coverage of the analysed articles, as well as on the approach to data interpretation.
- The ***Conclusions*** section has been revised and expanded in line with the reviewer's comment. It addresses the theoretical and practical implications of the study, outlines directions for future research building on the paper’s findings and discusses the broader limitations of the research.
